# Structural Plasticity of Flavin-Dependent Thymidylate Synthase Controlled by the Enzyme Redox State

**DOI:** 10.3390/biom15030318

**Published:** 2025-02-21

**Authors:** Ludovic Pecqueur, Murielle Lombard, Djemel Hamdane

**Affiliations:** Laboratoire de Chimie des Processus Biologiques, CNRS-UMR 8229, Collège De France, Université Pierre et Marie Curie, 11 place Marcelin Berthelot, CEDEX 05, 75231 Paris, Francemurielle.lombard@college-de-france.fr (M.L.)

**Keywords:** thymidylate synthase, ThyX, reduced FAD, conformational changes, FAD dynamic, X-ray crystallography

## Abstract

2′-Deoxythymidine-5′-monophosphate, dTMP, is an essential precursor of thymine, one of the four canonical bases of DNA. In almost all living organisms, dTMP is synthesized de novo by a reductive methylation reaction of 2′-deoxyuridine-5′-monophosphate (dUMP) catalyzed by the thymidylate synthase, where the carbon used for the methylation is derived from methylenetetrahydrofolate (CH2THF). Many microbes, including human pathogens, utilize the flavin-dependent thymidylate synthase encoded by the *thyX* gene to generate dTMP. The mechanism of action relies on the reduced coenzyme FADH^−^, which acts both as a mediator, facilitating methylene transfer from CH2THF to dUMP, and as a reducing agent. Here, we present for the first-time crystallographic structures of ThyX from *Thermotoga maritima* in the reduced state alone and in complex with dUMP. ThyX flavin reduction appears to order the active site, favoring a flavin conformation that drastically deviates from that observed in the oxidized enzyme. The structures show that FADH^−^ potentially controls access to the folate site and the conformation of two active site loops, affecting the degree of accessibility of substrate pockets to the solvent. Our results provide the molecular basis for the sequential enzyme mechanism implemented by ThyX during dTMP biosynthesis.

## 1. Introduction

**Figure 1:**
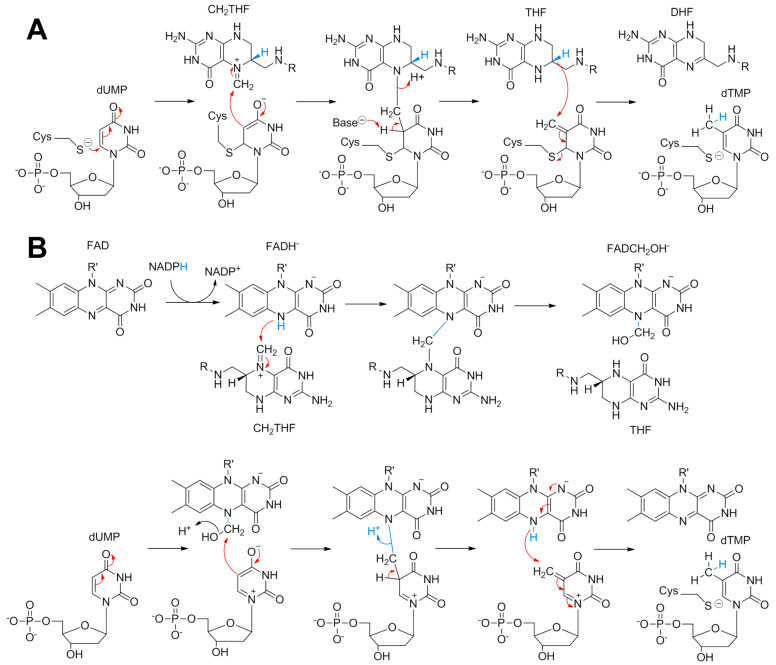
Postulated biosynthesis mechanism of thymidylate by the classical TS (**A**) and by FDTS, ThyX (**B**).

The reductive methylation of 2′-deoxyuridine-5′-monophosphate (dUMP) to 2′-deoxythymidine-5′-monophosphate (dTMP) is essential for cell replication and division. The resulting dTMP is the precursor of thymine, one of the canonical bases of DNA. All organisms rely on a thymidylate synthase (TS) to catalyze this vital reaction. To date, two completely different classes of TS have been discovered, and both enzymes retrieve the carbon source used for the methylation reaction from methylenetetrahydrofolate (CH2THF); the first being the classic TS found in the majority of living organisms including *Escherichia coli* with ThyA and humans with TYMS [1]. The latter is a major recognized target for cancer chemotherapies involving, for example, 5-fluorouridine. The molecular mechanism of classical TS has been firmly established (Figure 1A) through exhaustive structural and biochemical approaches using *E. coli* ThyA as a model [2].

The ThyA homodimer covalently activates the uracil C5 of dUMP via an active site cysteine prior to methylene acceptance from CH2THF [2]. Once the methylene has been transferred to the nucleotide, the tetrahydrofolate (THF) resulting from the first reaction step acts as a reducing agent of the exocyclic methylene to the methyl group, releasing dihydrofolate (DHF) and dTMP as the final products [2]. The alternative enzyme to ThyA is encoded by the *thyX* gene, the flavin-dependent TS (FDTS) found in over 30% of bacteria and in many archaea and viruses [3,4]. Since FDTS has a completely different structure and mechanism to that of classical TS [5,6,7], ThyX constitutes a serious antibacterial target [8,9]. The chemical mechanism of FDTS-dependent dTMP biosynthesis is highly complex, and has been the subject of numerous and varied mechanistic proposals [10,11,12,13,14,15]. Recently, Kohen’s group isolated a ThyX reaction intermediate in the form of a covalent FAD-CH2-dUMP complex, suggesting that flavin mediates methylene transfer between folate and dUMP [15], as observed in a flavin- and folate-dependent TrmFO tRNA methyltransferase involved in the reductive methylation of uridine 54 to ribothymidine [16,17]. Using a formaldehyde shunt, we recently obtained the structure of the dUMP methylating agent in ThyX in the form of a flavin carbinolamine [18], and consequently proposed an overall chemical mechanism of ThyX (Figure 1B). Activation of dUMP by polarization of the active site (and not by Michael addition of a cysteine as in the case of ThyA) enables the nucleophilic attack of C5-uracil on the electrophilic methylene of carbinolamine (Figure 1B). Once the methylene is transferred, it is reduced by the flavin hydroquinone to yield THF and dTMP. The strictly conserved active site residues, Tyr91 and Ser88, have been shown to be critical for activity through site-directed mutagenesis [13]. In our structural model of ThyX in complex with the FAD-carbinolamine intermediate, dUMP, and CH₂THF, we observed that the carbinolamine geometry favors an SN2-like attack by dUMP [18]. Additionally, in such a model, Ser88 and Tyr91 are positioned within hydrogen-bonding distance of the carbinolamine hydroxyl group. This suggests that these residues may facilitate the SN2 reaction by acting as acids, promoting the displacement of the β-hydroxyl leaving group (Figure 1B). Notably, the phenolic oxygen of Tyr91 is within hydrogen-bonding distance of the guanidinium nitrogen of Arg90, another conserved residue [13,18]. The proximity of this positively charged group could enhance the acidity of Tyr91, potentially making it a general acid catalyst that assists in the departure of the leaving water to give the covalent FAD-CH2-dUMP complex (Figure 1B). This hypothesis aligns with previous kinetic data showing that Y91 mutations in *T. maritima* ThyX cause a significant decrease in the rate constant for flavin carbinolamine consumption triggered by dUMP [13].

The structures of *T. maritima* ThyX in oxidized form (ThyX^ox^), notably that of the binary FAD/dUMP [5] and ternary CH2THF/FAD/dUMP complexes [19], have delineated the binding sites of the two substrates and further strengthened the chemical mechanism postulated in Figure 1B. However, these structures are not physiologically relevant since it has been shown that one of the first steps of the *T. maritima* FDTS reaction involves flavin reduction [20,21]; however, to date, no structure of reduced ThyX (ThyX^red^) is available. Here, we report the crystal structures of *T. maritima* ThyX^red^ alone and in the presence of dUMP as well as the crystal structures of the ThyX Y91F mutant with FAD in the oxidized and reduced form.

## 2. Materials and Methods

### 2.1. Chemicals

Chemicals were reagent grade and used as purchased without further purification, unless stated otherwise. 2′-Deoxyuridine 5′-monophosphate (dUMP) and reduced nicotinamide adenine dinucleotide phosphate (NADPH) were obtained from Sigma-Aldrich.

### 2.2. Site Directed Mutagenesis

The Y91F mutation was introduced using the QuickChange^®^ strategy and the following primers 5′-GAGCGGCAGATTCTCGAAGCTCTCCTACG-3′ (forward) and 5′ CGTAGGAGAGCTTCGAGAATCTGCCGCTC 3′ (reverse). PCR was performed with the Phusion DNA polymerase (ThermoFisher Scientific, Asnières-sur-Seine, France). Gene sequence was checked by DNA sequencing.

### 2.3. Protein Expression and Purification

ThyX *WT* (Uniprot ID: Q9WYT0) and Y91F from *T. maritima* were expressed in a pET11d transformed in BL21(DE3) using LB medium following induction with 0.5 mM isopropyl β-D-1-thiogalactopyranoside at an OD600 of 0.6. After overnight incubation at 29 °C, cells were harvested by centrifugation and lysed by sonication in 50 mM Tris pH 8, 150 mM NaCl, 1 mM β-mercaptoethanol, and 1 mM phenylmethanesulfonyl fluoride. The cleared lysate was purified in batch mode on a Ni-NTA column (8 mL). After loading, the column was washed with 150 mL Tris 50 mM pH 8, NaCl 150 mM, and imidazole 20 mM. The protein was eluted with five elution steps of 8 mL of Tris 50 mM pH 8, NaCl 150 mM, and imidazole 250 mM. Fractions containing the protein were pooled and concentrated by ultrafiltration with an Amicon Ultra-15 (cut-off 30 kDa, Millipore, Guyancourt, France). The sample was further purified by size-exclusion chromatography using a S200 16/60 equilibrated in Tris 50 mM pH 7.5 and NaCl 150 mM. Protein purity was checked by sodium dodecyl sulfate-polyacrylamide gel electrophoresis. Protein concentration was estimated by the Bradford method [22] using BSA as a standard.

### 2.4. ThyX-Flavin Reconstitution

ThyX was reconstituted with flavins as detailed previously by incubating 100 µM FDTS with 1 mM of FAD for 30 min at 25 °C. Excess flavin was removed with an Illustra NAP-25 column equilibrated in Tris 50 mM pH 7.4 and NaCl 150 mM (buffer A). The protein was concentrated with a Vivaspin-500 (cut-off 30 kDa, Sartorius). Residual unbound flavin found in the filtrate was removed by cycles of dilution/concentration with buffer A.

### 2.5. Crystallization and Data Collection

All crystals were obtained by the vapor diffusion method in a glove box with <1.3 p.p.m. O2. Crystals were grown by mixing 1 µL of protein at 5–7 mg·mL^−1^ in Tris 50 mM pH 7.4, NaCl 150 mM with 1 µL of precipitant solution consisting of 42–46% *w/v* PEG 200 in Tris 0.1 M pH 7.5.

To obtain crystals of ThyX^red^, a solution of 5–7 mg·mL^−1^ of reconstituted ThyX^ox^, degassed overnight in a glovebox, was first reduced in Tris 50 mM pH 7.4 NaCl 150 mM by adding stepwise an excess of dithionite (1.3 eq). The reduction reaction was followed in the glovebox by UV–Visible absorption spectroscopy using a Jasco V750 spectrophotometer coupled to optic fibers. The resulting solution was immediately used for crystallization, as described above. Crystals of ThyX^red^ were grown overnight and flash frozen the next day. To obtain the complex with dUMP, crystals were soaked for 5–10 min in a reservoir solution supplemented with 5 mM dUMP. All crystals were directly flash frozen in liquid propane.

All diffraction data were collected at 100 K on single crystals at the beamlines Proxima-1 and Proxima-2 at the SOLEIL synchrotron (Saint-Aubin, France). Data were indexed, processed, and scaled using autoPROC [23]. Structure refinement was performed with autoBUSTER [24]. Data processing and refinement statistics are summarized in Table S1. Ligand–protein interaction diagrams were obtained from LigPlot+ [25].

## 3. Results and Discussion

**Figure 2:**
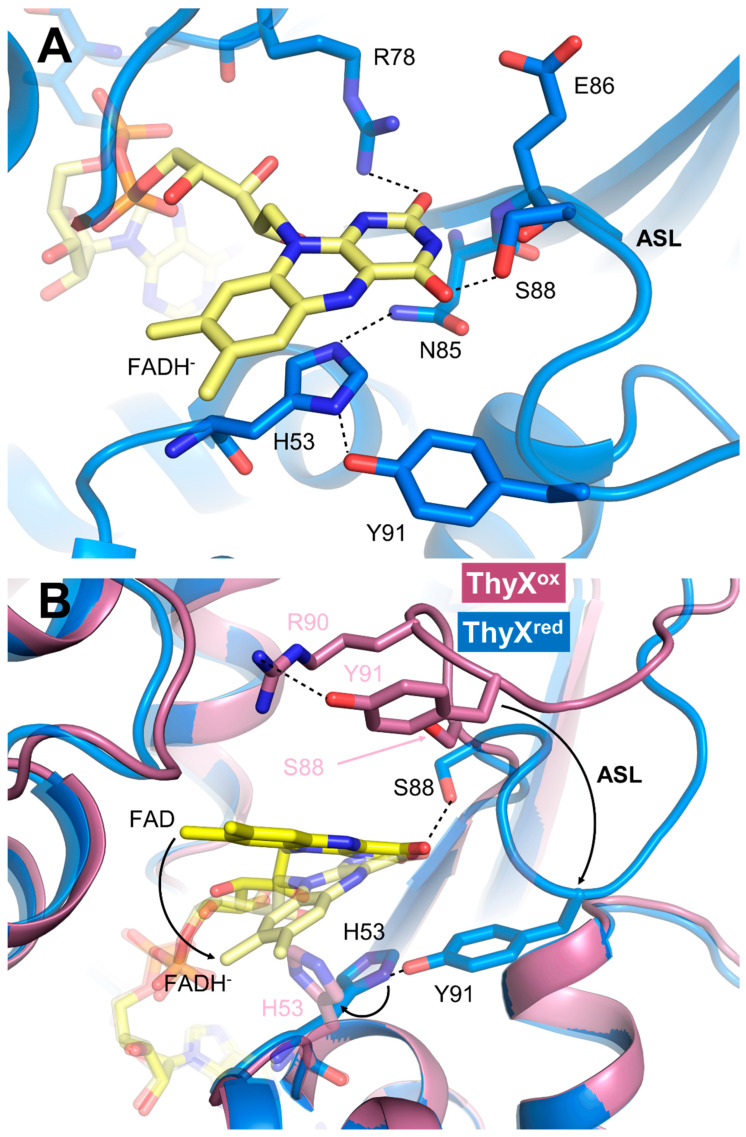
Structure of the *T. maritima* ThyX active site in the reduced form. (**A**) Section of the ThyX^red^ active site showing the FADH^−^ prosthetic group (yellow) and the residues interacting with the isoalloxazine moiety. The dotted lines represent the hydrogen bonds between the atoms involved in this type of interaction. (**B**) Superposition of the active site structure of ThyX^red^ (blue) with that of ThyX^ox^ (PDB: 1O2A, pink). The oxidized flavin, FAD, in ThyX^ox^ is shown as a sandy yellow ball-stick, while that of FADH^−^ in ThyX^red^ is in yellow. The arrows show the repositioning trajectory of the flavin coenzyme and the active site loop (ASL) carrying the Y91 residue in the course of the enzyme reduction. The dashed portion of the ASL in ThyX^ox^ indicates the weak electron density for this region, suggesting that this loop is flexible under the oxidized state.

The ThyX^red^ structure was obtained by prior reduction of the oxidized enzyme with an excess of dithionite under anaerobic conditions (Figure S1). The ThyX^red^ structure was determined at 2 Å resolution in the space group P212121, with four molecules in the asymmetric unit (Table S1). As expected, the enzyme formed a tetramer where four identical active sites forming large cavities were generated at the interface of three different monomers (Figure S2A). The general fold was preserved, and no major changes were observed compared with ThyX^ox^, as evidenced by the low RMSD of 0.41 ± 0.07 Å over 206 Cα (Figure S2B, Table S2). However, significant changes were identified in the active site of the enzyme containing the flavin coenzyme (Figure 2A).

**Figure 3:**
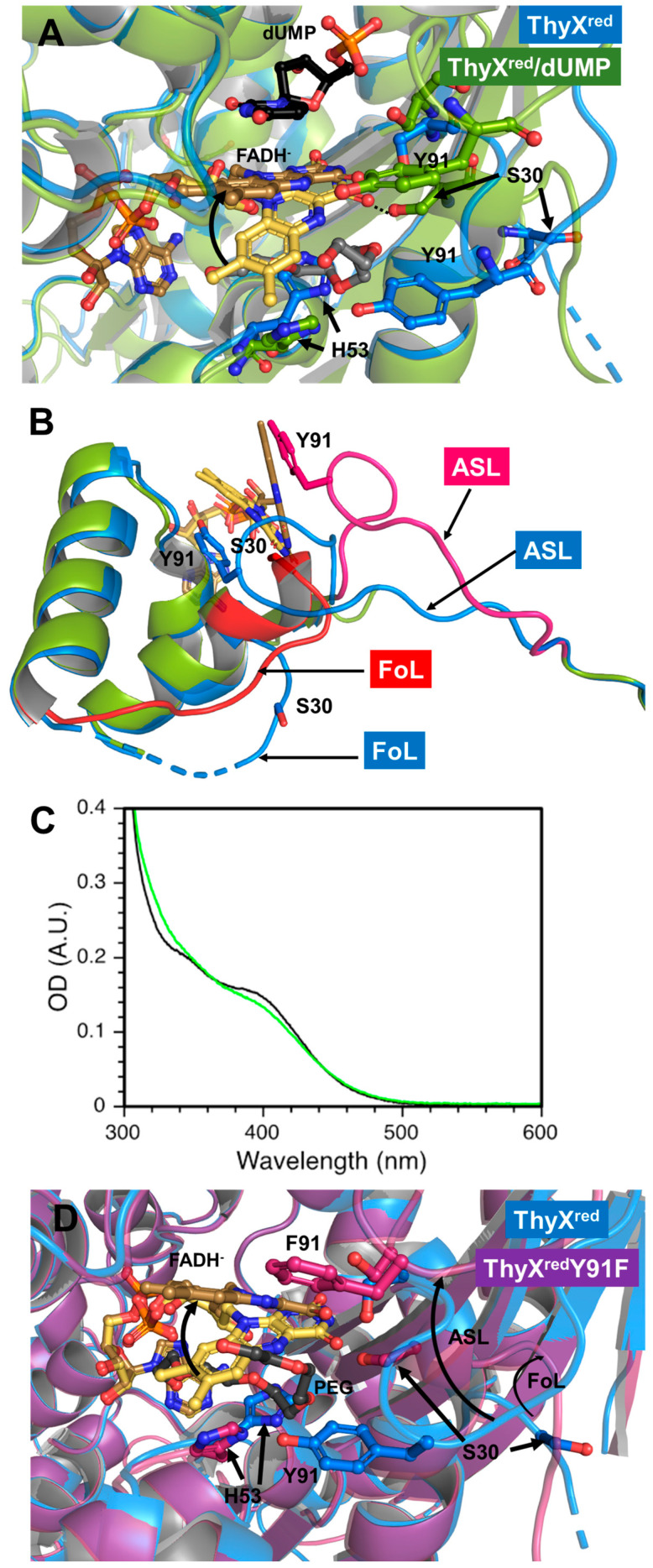
Structure of the *T. maritima* ThyX^red^ active site in complex with dUMP and the effect of hydrogen bond disruption between H53 and Y91 in the Y91F mutant on the structure of the active site. (**A**) Superposition of the active site structure of ThyX^red^/dUMP (green) with that of ThyX^red^ (blue). The FADH- prosthetic groups are colored in sand and yellow for ThyX^red^/dUMP and ThyX^red^, respectively. The dotted lines represent the hydrogen bonds between the C=O4 carbonyl of FADH- and the side chain of S30. The dUMP substrate (black) lies on the re-face of the isoalloxazine. The PEG molecule lying within the folate cavity is represented as gray sticks. The arrows show the repositioning trajectory of the flavin coenzyme and residues of the ThyX active site following dUMP binding. (**B**) Conformational changes in two loops of the *T. maritima* ThyX^red^ active site, namely ASL and FoL, upon dUMP binding. The ASL and FoL loops in the ThyX^red^/dUMP complex are shown in magenta and red, respectively. (**C**) UV/Vis absorption spectrum of reduced flavin in the ThyX wild type (black, 54 µM ThyX) and Y91F mutant (green). (**D**) Structure of the *T. maritima* ThyX^red^ Y91F active site. Superposition of the active site structure of ThyX^red^ Y91F (purple) with that of ThyX^red^ (blue). The FADH- prosthetic groups are colored in sand and yellow for ThyX^red^ Y91F and ThyX^red^, respectively. The PEG molecule lying within the folate cavity of ThyX^red^ Y91F is represented as gray sticks. The arrows show the repositioning trajectory of the flavin coenzyme, residues of the ThyX active site, and the ASL/FoL loops upon Y91F mutation.

The FAD isoalloxazine adopts a butterfly conformation with a dihedral angle of 13°, consistent with a reduced flavin state. Moreover, the reduced flavin is probably under the anionic hydroquinone form (FADH^−^), as a conserved arginine (R78) points its side-chain guanidinium group toward the N1 and C=O2 of the isoalloxazine to stabilize the delocalized negative charge of this hydroquinone species. FADH^−^ is frequently stabilized via hydrogen bonding by a basic residue facing its N1 in several types of flavoproteins [26,27]. Remarkably, the position of the isoalloxazine in the active site differs between FAD and FADH^−^ (Figure 2B). For comparison of the changes produced, we provide the difference in distance between the C8 of isoalloxazine FADH^−^ and FAD, which is d(C8FADH–C8FAD) ~5 Å. In the case of ThyX^red^, the isoalloxazine is repositioned toward the bottom of the active site pocket, so that it approaches the helix-H3 of the monomer-3 carrying the conserved histidine H53, and is stabilized by a series of interactions with the polypeptide (Figure 2A). The imidazole of this histidine is engaged in a π–π interaction with the benzyl ring of the isoalloxazine FADH^−^ si-face and is firmly held in place by two H bonds involving its two nitrogens with the side chains of Y91 and N85. In the ThyX^ox^ CH2THF/FAD/dUMP structure, this same histidine enters in π–π interaction with the pteridine of folate, stabilizing the stacking between folate and isoalloxazine [19]. Of note, H53 has been shown to present weak electron density and various conformations in several ThyX^ox^ structures [5,6,19]. The intervention of a histidine as a folate stabilizing agent is not unique to ThyX and has also been observed in the structure of the flavoenzyme TrmFO in complex with THF [28]. Besides the interaction between FADH^−^ and R78, several other interactions stabilize the pyrimidine moiety of the isoalloxazine of FADH^−^, notably (i) the main amide group of E86 hydrogen bonds with the C=O2, (ii) the carbonyl of the same residue interacts with the NH3, and (iii) while the side chain of S88 engages an H bond with C=O4 of FAD (Figure 2A and Figure S3). In this configuration, the isoalloxazine in ThyX^red^ appears to partially occupy the CH2THF binding site, making it impossible for the folate to access its binding site. On the other hand, a huge cavity accessible to the solvent on the proximal side is created where the dUMP binding site is located (Figure S4). In the ThyX^ox^ structure, the dUMP cavity is not accessible to the solvent, requiring a change in conformation of the active site loop (ASL, 86 to 97), which is flexible (Figure 2B). It is interesting to note that in ThyX^red^, the phenol group of the conserved Y91 contained in the ASL establishes a hydrogen bond with the imidazole of H53, thereby maintaining an extended version of the ASL, which no longer appears flexible (Figure 2B). Thus, this structure of ThyX^red^ reveals that, due to the positioning of the reduced coenzyme and the ASL, dUMP is the first substrate to bind to the reduced enzyme, in agreement with previously published kinetic data [20,21]. To examine this in a little more detail, we have made the structure of ThyX^red^ in complex with dUMP by co-crystallization, which diffracts at ~2 Å in the same space group as the reduced enzyme in the absence of substrates (Table S1). The binding of dUMP appears to have reorganized the active site by relocating the flavin in the canonical configuration observed in all ThyX^ox^ structures, wherein the re-face of the reduced isoalloxazine is in π–π interaction with the uracil of dUMP (Figure 3A and Figure S5).

The imidazole of H53 undergoes a drastic 75° reorientation toward the solvent compared with that observed in ThyX^red^ and no longer interacts with FAD (Figure 3A). This conformational change liberates the folate cavity, allowing a PEG molecule to occupy it. The interaction between FADH^−^ and uracil appears to be responsible for the constrained planarity of the isoalloxazine, reducing the dihedral angle to roughly 1° (quasi-planar). We have recently shown that the addition of dUMP to ThyX^red^ alters the UV/Visible absorption spectrum of the reduced flavin, which is redshifted, consistent with a change in the flavin environment upon dUMP binding [29]. Furthermore, based on our femtosecond transient absorption studies, we proposed that dUMP binding to ThyX^red^ appears to decrease the distribution of ground-state configurations and exert a constraint on the butterfly bending motion of isoalloxazine in the excited state. These results agreed well with a close stacking of dUMP to the reduced isoalloxazine, and were therefore in perfect agreement with our ThyX^red^ and Thy^red^/dUMP structures. Additional changes were also apparent in the ASL and another loop in the immediate vicinity, referred to as the folate loop (FoL, 27–37), as it restricts access to folate. FoL was shortened by the formation of an additional helix turn of helix-H1 directly at its N-terminus, enabling S30 to engage in hydrogen bonding with the C=O4 of FADH^−^ instead of S88 in ThyX^red^ (Figure 3B). Similarly, the ASL tended to retract, disrupting the hydrogen bond between Y91 and H53. This structural change isolated dUMP from the solvent and cleared the way for sequential binding of the carbon donor (Figure 3A,B).

As mentioned, the interaction between Y91 and H53 could be a key factor in the closed conformation of the folate site. To investigate this further, we generated the Y91F ThyX mutant and characterized it. The Y91F mutant has a flavin content similar to that of the WT, but with different absorption spectra of the reduced forms, indicating an altered flavin environment in the mutant (Figure 3C). In addition, mutation of this Y91 has been shown to induce a 1000-fold decrease in flavin oxidation rate during the methylation reaction compared with the WT [13]. In order to obtain the molecular basis for the difference in FADH^−^ environment in the mutant, the structure of ThyX^red^ Y91F was obtained (Table S1). Surprisingly, ThyX^red^ Y91F showed that the active site differed from that of the WT in the positioning of the FADH^−^ and the conformation of both the ASL and FoL loops (Figure 3D). The absence of the H-bond seemed to favor an organization of the active site like that observed in ThyX^ox^. Thus, the H53-Y91 interaction could indeed constitute an important stabilizing feature of the flavin’s ‘OFF’ conformation.

## 4. Conclusions

In conclusion, our structural investigation has provided the first structures of ThyX^red^, enabling a better understanding of the enzymatic mechanism of this FDTS. These structures offer a molecular basis for the kinetic and biochemical results published on *T. maritima* ThyX. Based on the NADPH oxidase activity of *T. maritima* ThyX, an enzymatic model was proposed in which the reductive methylation reaction begins with the formation of reduced flavin (here, we propose FADH^−^; structure of ThyX^red^) following the reaction of FAD with NADPH. The reduced enzyme then binds dUMP (structure of ThyX^red^ FADH^−^/dUMP) and folate. The ThyX^red^ structure explains why this sequential order of substrate binding by ThyX is made possible, which is mainly through the fact that FADH^−^ is stabilized in an ‘OFF’ conformation that obstructs access to the folate site while opening wide the dUMP-binding cavity (Figure 3D). Once the dUMP site is filled, the folate site is released by switching FADH^−^ to a position compatible for mediating methylene transfer from folate to dUMP (‘ON’ conformation) and by the coordinated conformational change of the two loops, namely ASL and FoL. We propose that the redox state of FDTS may govern the plasticity of the enzyme’s active site, and hence the order of substrate selection. These results could pave the way for the development of selective ThyX inhibitors based on the enzyme’s redox state.

## Data Availability

Raw data collection and refinement for the X-ray structures of the ThyX WT and Y91F mutant (PDB: 8REN, 8REO, 8REP, 8REQ) are available in the PDB. The data presented in this study are available in the Protein Data Bank at https://www.rcsb.org/ under the reference numbers 8REN, 8REO, 8REP, and 8REQ.

## Supplementary Materials

The following supporting information can be downloaded at: https://www.mdpi.com/article/10.3390/biom15030318/s1, Figure S1: Absorption spectrum of ThyX^ox^ before and after reduction with dithionite. Figure S2: Comparison of the reduced and oxidized ThyX*wt*. Figure S3: Environment of the flavin in ThyX*wt* in the reduced state, the oxidized state and in the reduced state in complex with dUMP. Figure S4: Changes in the surface of ThyX upon FAD reduction, dUMP and folate binding. Figure S5: Conformational changes in the active site of ThyX upon FAD reduction, dUMP and folate binding. Table S1: Summary of data collection and refinement statistics, Table S2: r.m.s.d. are calculated from the superposition of 206 Cα.
