# Supplementary material for: Structural Plasticity of Flavin-Dependent Thymidylate Synthase Controlled by the Enzyme Redox State
Source: Biomolecules. 2025 Feb 21;15(3):318. doi: 10.3390/biom15030318 (PMC11940539; doi:10.3390/biom15030318)
Supplement: Supplementary file 1 [file biomolecules-15-00318-s001.zip › biomolecules-3447149-supplementary.pdf]

Structural Plasticity of Flavin-Dependent Thymidylate

Synthase Controlled by the Enzyme Redox State

Ludovic Pecqueur, Murielle Lombard and Djemel Hamdane *,†

Laboratoire de Chimie des Processus Biologiques, CNRS-UMR 8229, Collège De France, Université Pierre et Marie Curie, 11 place Marcelin Berthelot, CEDEX 05, 75231 Paris, France; murielle.lombard@college-de-france.fr (M.L.)

* Correspondence: djemel.hamdane@sorbonne-universite.fr

† Current address: Laboratoire Développement, Adaptation et Vieillissement Dev2A, IBPS, Sorbonne Université-CNRS UMR8263, Inserm U1345, Sorbonne Université 7 quai Saint Bernard, CEDEX 05, 75252 Paris, France.

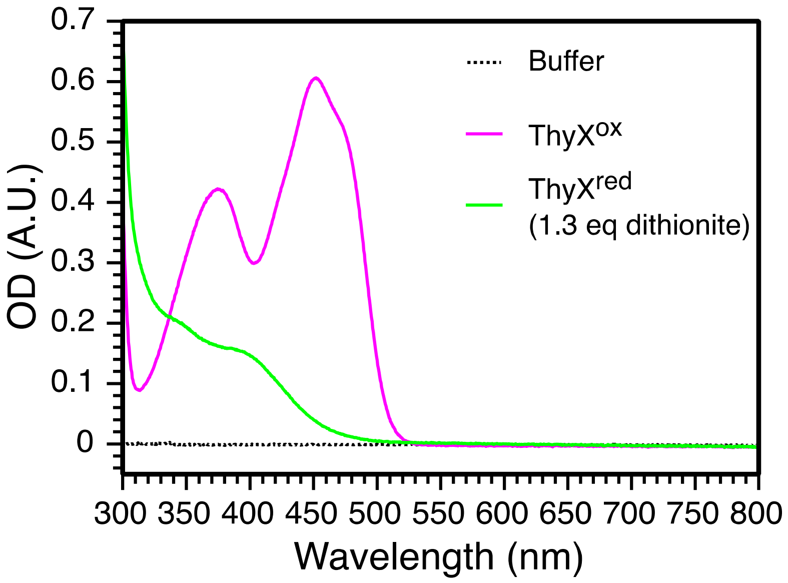

Figure S1: Absorption spectrum of ThyX^{ox} before and after reduction with dithionite. In a glovebox with <0.5 p.p.m. O_2, 50 µM of ThyX^{ox} in Tris 50 mM pH 7.4, NaCl 150 mM was reduced with an excess of dithionite.

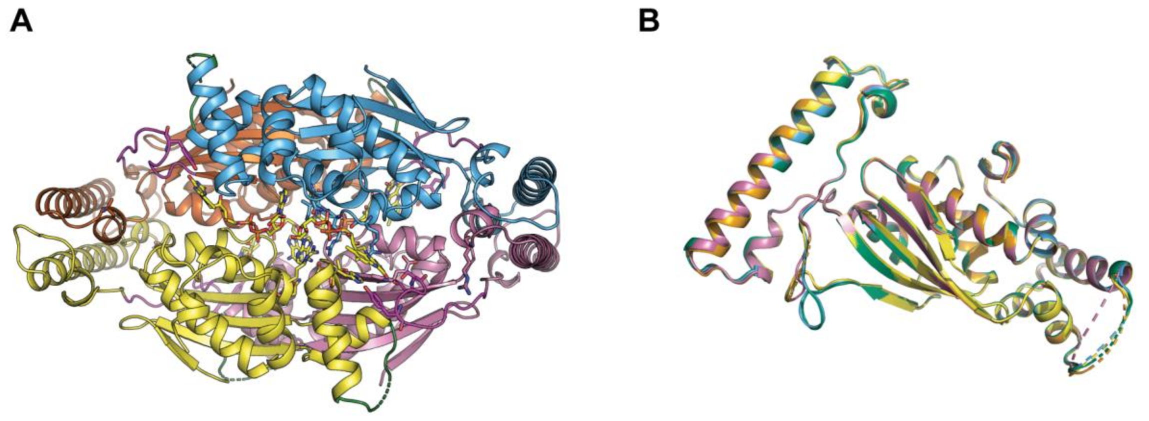

Figure S2: Comparison of the reduced and oxidized ThyX*wt* (ThyX^{red} chain A in green vs ThyX^{ox} chains A to D in 1O2A colored yellow, magenta, orange, blue respectively)

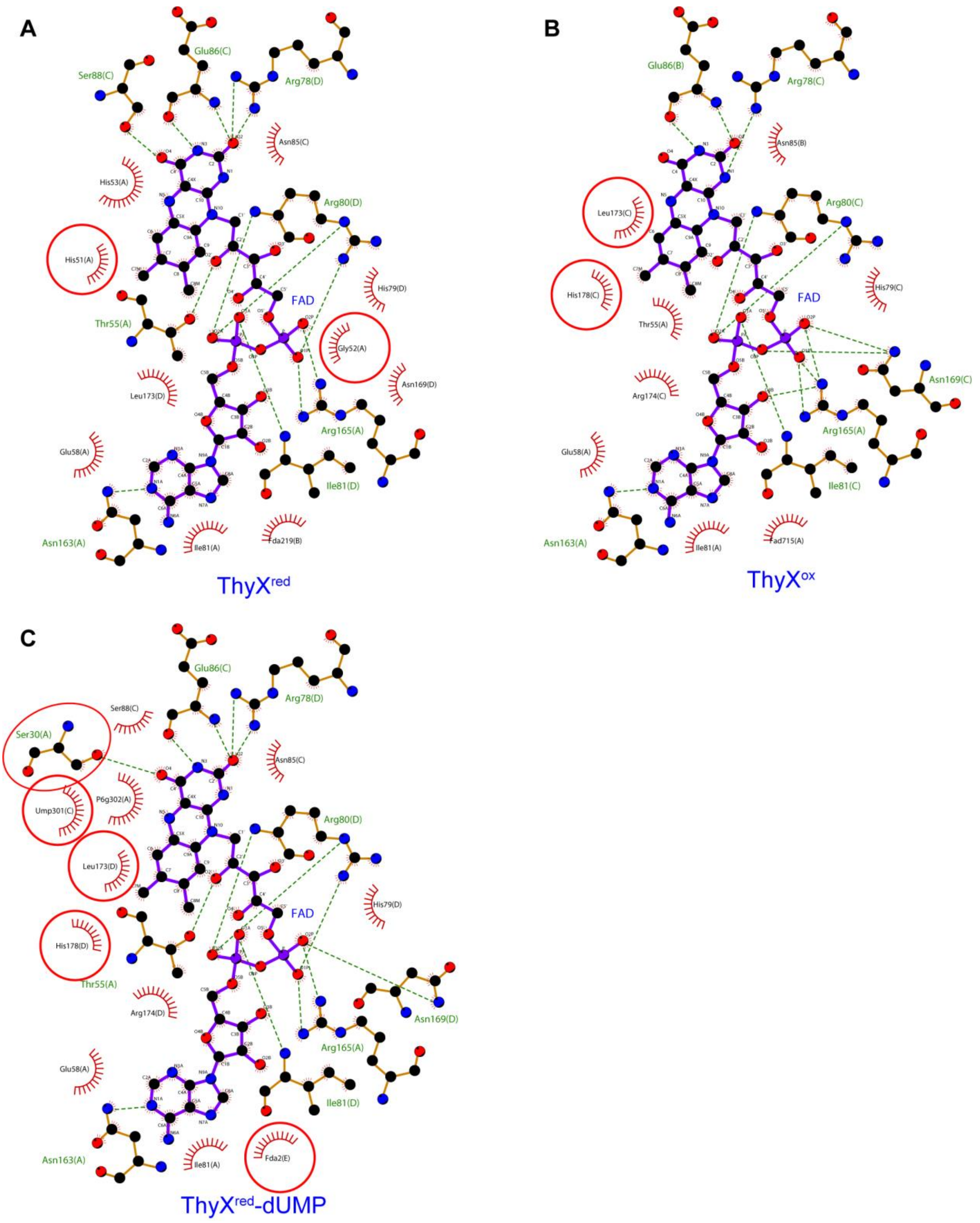

Figure S3: Environment of the flavin in ThyX*wt* in the reduced state (A), the oxidized state (B) and in the reduced state in complex with dUMP (C). Nonequivalent residues are circled in red. Figures were generated with LigPlot v2.2.8 (4)

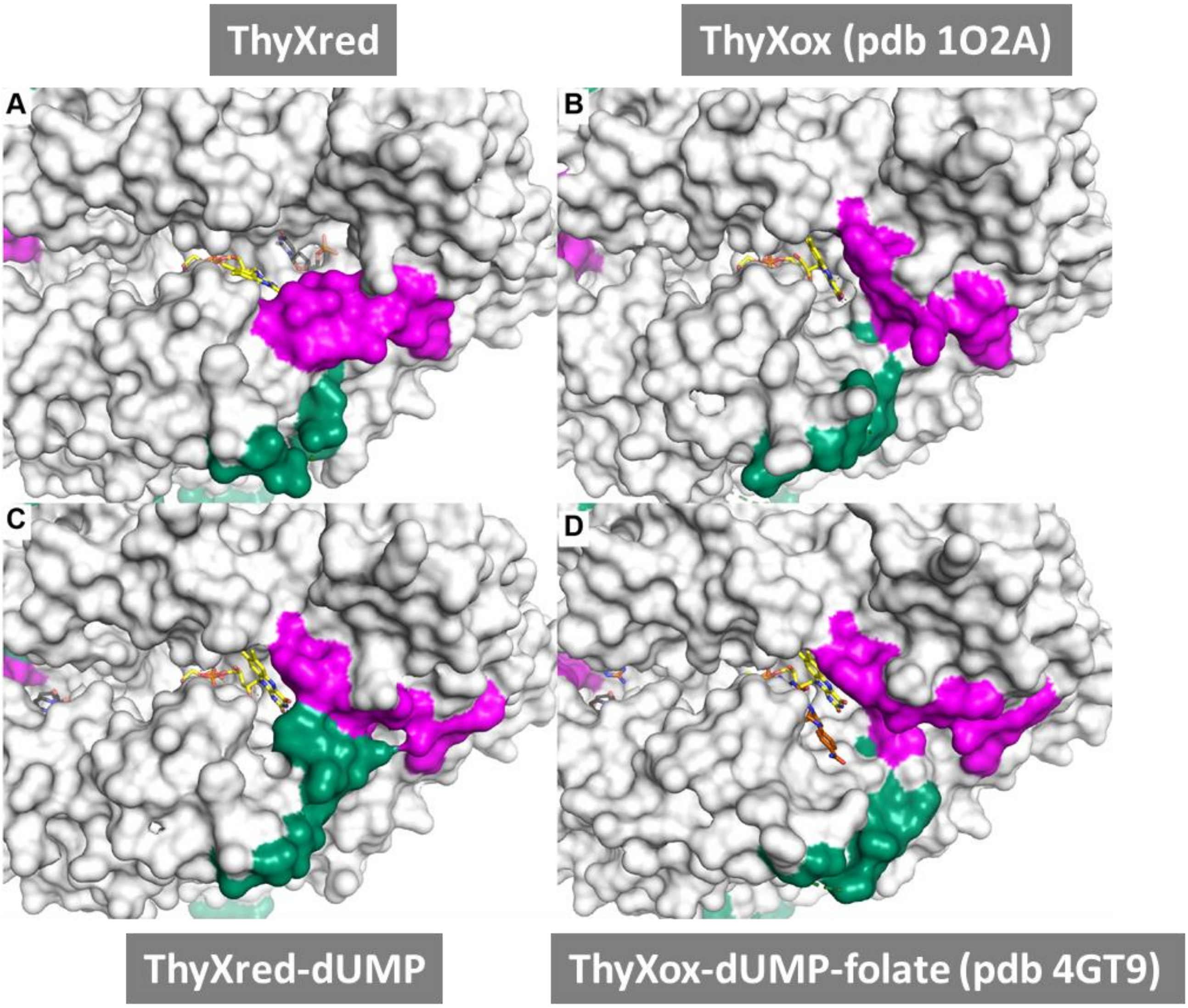

Figure S4: Changes in the surface of ThyX upon FAD reduction, dUMP and folate binding. A) Surface of ThyX^{red}, B) ThyX^{ox} (pdb 1O2A), C) ThyX^{red}-dUMP, D) ThyX^{ox}-dUMP-folate (pdb 4GT9). The active site loop (residues 86-97) is colored in magenta and the folate loop is colored in green. In A), a dUMP molecule (black) from the structure of ThyX^{red}-dUMP is shown as transparent sticks for easy visualization of the dUMP binding site. The FAD and folate are in yellow and orange sticks, respectively.

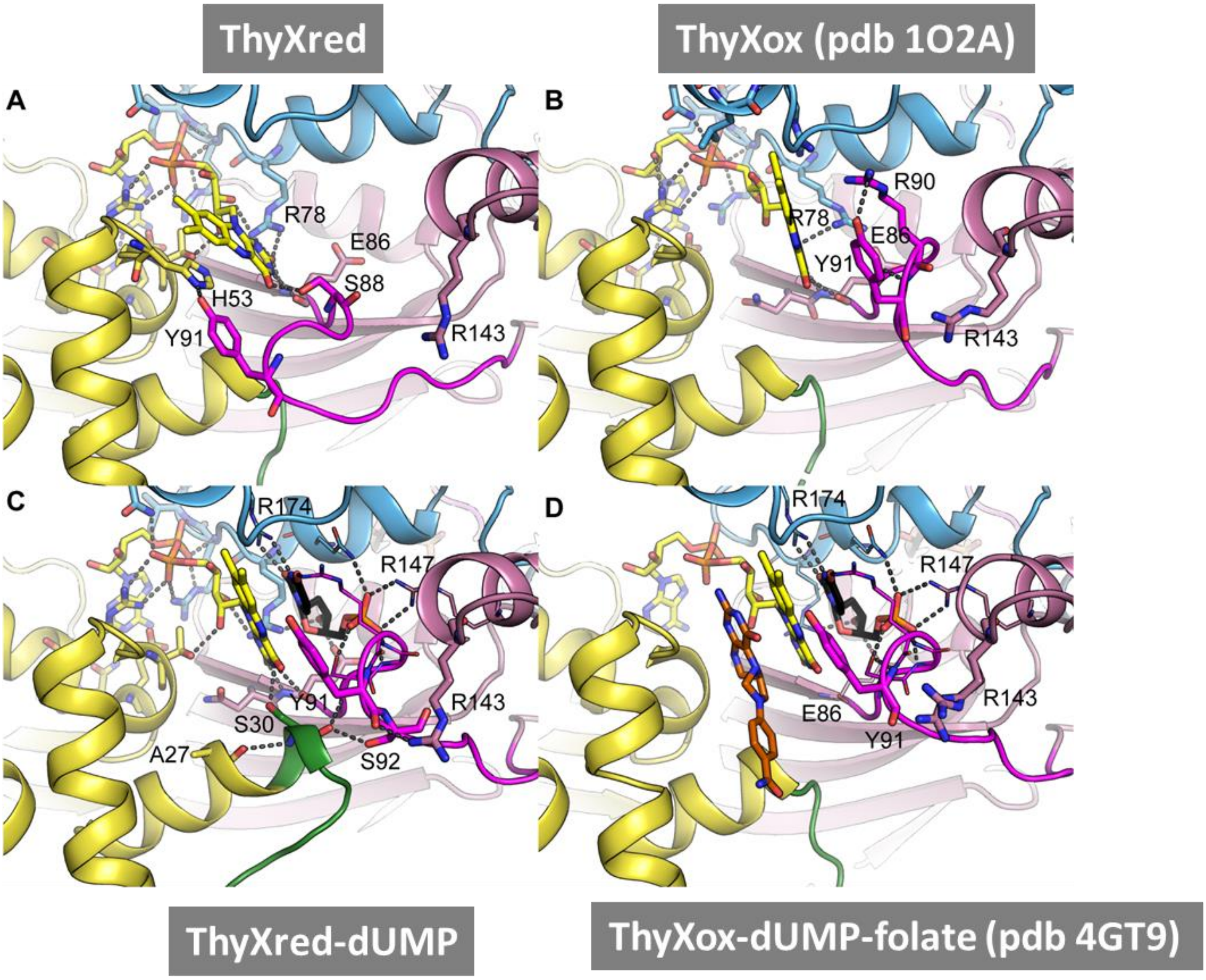

Figure S5: Conformational changes in the active site of ThyX upon FAD reduction, dUMP and folate binding. A) $ThyX^{red}$, B) $ThyX^{ox}$ (pdb 1O2A), C) $ThyX^{red}$-dUMP, D) $ThyX^{ox}$-dUMP-folate (pdb 4GT9). The carbons of dUMP and the folate are colored in black and orange, respectively. Monomers are colored in yellow, pink and blue. The active site loop (residues 86-97) is colored in magenta and the folate loop is colored in green. Dashed lines in grey represent potential hydrogen bonds. Residues shown as lines interact with the substrate dUMP.

Table S1- Summary of data collection and refinement statistics

	ThyX^{red}	ThyX^{red}-dUMP	ThyX^{ox}-Y91F	ThyX^{red}-Y91F
**Data collection**				
Beamline	Soleil Proxima 1	Soleil Proxima 1	Soleil Proxima 2	Soleil Proxima 1
Photon energy (keV)	12.67	12.67	12.65	12.67
Space group	$P\ 2_1\ 2_1\ 2_1$	$P\ 2_1\ 2_1\ 2_1$	$P\ 2_1\ 2_1\ 2_1$	$P\ 2_1\ 2_1\ 2_1$
Cell dimensions				
a, *b*, *c* (Å)	53.68, 116.61, 140.57	54.09, 115.96, 140.53	54.92, 116.80, 142.75	55.02, 117.09, 141.97
α, β, γ (°)	90, 90, 90	90, 90, 90	90, 90, 90	90, 90, 90
Resolution (Å)	89.75-2.139 (2.39-2.14)	89.44-2.03 (2.24-2.03)	90.40-2.20 (2.24-2.20)	90.33-1.94 (1.97-1.94)
R_{sym} or R_{merge}	0.097 (1.241)	0.120 (1.433)	0.232 (2.403)	0.096 (2.711)
R_{meas}	0.104 (1.323)	0.128 (1.521)	0.265 (2.607)	0.104 (2.988)
$I / \sigma I$	11.8 (1.5)	10.1 (1.5)	5.6 (1.1)	9.3 (0.6)
Completeness (%)				
spherical	61.8 (11.2)	63.2 (12.5)	99.7 (100)	100 (100)
ellipsoidal	93.4 (78.5)	93.6 (68.6)	-	-
No. unique reflections	30639 (1532)	36654 (1834)	47316 (2301)	68746 (3394)
Redundancy	7.6 (8.4)	8.5 (9.0)	6.5 (6.1)	6.7 (5.7)
CC(1/2)	0.999 (0.653)	0.999 (0.660)	0.990 (0.347)	0.999 (0.370)
**Refinement**				
Resolution (Å)	36.52-2.14	38.07-2.03	43.53-2.20	38.58-1.94
No. reflections	30627	36642	46240	68412
R_{work} / R_{free}	0.220/0.251	0.210/0.236	0.222/0.241	0.213/0.240
No. atoms				
Protein	7015	7090	6987	7109
Ligand/ion	249	362	312	388
Water	74	107	99	184
B-factors				
Macromolecule	49.8	41.7	42.0	46.9
flavin	44.2	35.8	35.2	38.1
UMP or FdUMP	-	36.3	-	-
PEG	59.6	57.4	57.3	65.9
Water	43.9	37.9	39.6	52.1
R.m.s. deviations				
Bond lengths (Å)	0.006	0.006	0.007	0.008
Bond angles (°)	0.83	0.87	0.88	0.90
Ramachandran				
Favored (%)	97.76	98.15	97.98	97.67
Allowed (%)	2.24	1.85	2.02	2.21
Outliers (%)	0.00	0.00	0.00	0.12
Molprobity Clashscore	2.64	3.28	3.35	4.12
PDB id	8REN	8REO	8REP	8REQ

Values in parenthesis correspond to the last resolution shell

Table S2. r.m.s.d. are calculated from the superposition of 206 Cα. FAD^{ox} corresponds to the coordinates deposited with the pdb code 1O2A. r.m.s.d. were calculated using the program ProFit (http://www.bioinf.org.uk/software/profit/)

	FAD^{ox} chain A	FAD^{ox} chain B	FAD^{ox} chain C	FAD^{ox} chain D
FAD^{red} chain A	0.465	0.470	0.474	0.261
FAD^{red} chain B	0.417	0.558	0.452	0.418
FAD^{red} chain C	0.403	0.471	0.357	0.333
FAD^{red} chain D	0.424	0.408	0.394	0.328
